# Geographical Scale Effects on the Analysis of Leptospirosis Determinants

**DOI:** 10.3390/ijerph111010366

**Published:** 2014-10-10

**Authors:** Renata Gracie, Christovam Barcellos, Mônica Magalhães, Reinaldo Souza-Santos, Paulo Rubens Guimarães Barrocas

**Affiliations:** 1 FIOCRUZ, ICICT/LIS, Núcleo de Geoprocessamento, Avenida Brasil, 4365 Pavilhão Haity Moussatché, sala 231, Rio de Janeiro 21045-900, Brazil; 2 FIOCRUZ, ICICT/LIS, Núcleo de Geoprocessamento, Avenida Brasil, 4365 Pavilhão Hai ty Moussatché, sala 231- Manguinhos, Rio de Janeiro 21045-900, Brazil; E-Mails: xris@fiocruz.br (C.B.); monica.magalhaes@icict.fiocruz.br (M.M.); 3 FIOCRUZ, ENSP/DENSP, Rua Leopoldo Bulhões, 1480 ENSP, sala 607—Manguinhos, Rio de Janeiro, ENSP 21041-210, Brazil; E-Mail: rssantos@ensp.fiocruz.br; 4 FIOCRUZ, ENSP/DSSA, Rua Leopoldo Bulhões, 1480 ENSP, sala 521—Manguinhos, Rio de Janeiro 21041-210, Brazil; E-Mail: paulo.barrocas@ensp.fiocruz.br

**Keywords:** leptospirosis, geographical scale, unit of analysis, socioeconomic indicators, environmental indicators

## Abstract

Leptospirosis displays a great diversity of routes of exposure, reservoirs, etiologic agents, and clinical symptoms. It occurs almost worldwide but its pattern of transmission varies depending where it happens. Climate change may increase the number of cases, especially in developing countries, like Brazil. Spatial analysis studies of leptospirosis have highlighted the importance of socioeconomic and environmental context. Hence, the choice of the geographical scale and unit of analysis used in the studies is pivotal, because it restricts the indicators available for the analysis and may bias the results. In this study, we evaluated which environmental and socioeconomic factors, typically used to characterize the risks of leptospirosis transmission, are more relevant at different geographical scales (*i.e.*, regional, municipal, and local). Geographic Information Systems were used for data analysis. Correlations between leptospirosis incidence and several socioeconomic and environmental indicators were calculated at different geographical scales. At the regional scale, the strongest correlations were observed between leptospirosis incidence and the amount of people living in slums, or the percent of the area densely urbanized. At the municipal scale, there were no significant correlations. At the local level, the percent of the area prone to flooding best correlated with leptospirosis incidence.

## 1. Introduction

Leptospirosis is a multifactorial disease that is well correlated with environmental (e.g., rainfall regime, temperature, topography, *etc.*), as well as, socioeconomic factors (e.g., sanitation conditions, population education, land use, *etc.*). The form and intensity of leptospirosis transmission can vary due to its diversity of reservoirs and serovars. Indeed, although it occurs almost worldwide, leptospirosis transmission patterns vary as a function of the region where it arises [1]. In developed countries, the transmission is limited, and usually associated with animal husbandry or some occupational activities, such as when workers are be exposed to contaminated waters [2,3,4,5].

On the other hand, leptospirosis occurrence in developing countries is related with intense and rapid urbanization without adequate infrastructure, resulting in sanitation problems, especially in poor vulnerable areas (*i.e.*, slums) located close to rivers or channels, prone to periodical flooding. In addition, we can distinguish different transmission patterns when comparing rural and urban areas, which may show endemic or epidemic patterns [4,5].

Climate change may increase the number of leptospirosis cases, mainly in developing countries, like Brazil [6]. In fact, it was observed an increase in the number of leptospirosis cases in Brazil from 1999 to 2011 was observed. In the first years of this period, there was an average of 2987 notifiable cases per year. In contrast, by the end of it, an average of 3942 cases per year was reported, which represented a raise of more than 24%, with an average mortality rate of 10% [7].

Thus, the results of leptospirosis risk factor studies are influenced by the period (*i.e.*, endemic or epidemic), environment (*i.e.*, urban or rural), and geographical scale (*i.e.*, global, regional, and local) considered. For example, several studies have been conducted in Brazil and other parts of the world, using different geographical scales, producing distinct results. Most of the research has been done in urban areas, mainly slums, followed by rural and wilderness areas [2,3,8,9,10,11,12,13,14].

These spatial designs, used by the researchers, affect the results obtained because each space scale used has its own attributes, while, some of them can be transferred to another space scale by specific data transformation methods (e.g., aggregation, generalization, simplification and selection). Hence, the degree of information detail depends on the chosen scale of geographical analysis. This choice is not arbitrary, it is designed to best achieve the study’s objectives, and necessarily means that only part of the information will be analyzed, since it is impossible to consider all existing elements at a given geographical scale. The identification of the research question leads to the recognition of which geographical elements should be either included or excluded from the study [15].

The objective of this study was to assess the relationships among various environmental and socioeconomical factors and leptospirosis incidence in Rio de Janeiro state, Brazil, from 1996 to 1999, using different geographical scales and units of analysis. Our hypothesis was that the associations observed were biased by the scales and units chosen. Therefore, the choice of scale is critical to design proper surveillance programs of this disease.

## 2. Materials and Methods

### 2.1. Geographical Scales and Units of Data Aggregation

To identify and discuss the environmental and socioeconomic determinants associated with the occurrence of leptospirosis at different geographical scales, three scales were chosen for the study, considering the political hierarchical divisions of the Brazilian Federative Republic: a *state level*, in which the health surveillance is performed by the state health secretary; a *municipal level*, where the occurrence of diseases are monitored by the municipal health secretary; and a *local level*, where the basic health units managers have to report the incidence of notifiable diseases to the municipal health secretary. Brazil is divided into 26 states, each of them is divided into municipalities. Each state is ruled by an elected governor, while elected mayors run municipalities. A municipality is divided into administrative regions (RA), neighborhoods and census sectors. From a census perspective, the smallest unit of aggregation of data is a census sector, where socioeconomic data is surveyed. Thus, for this study, at the *state level*, all 92 municipalities located in the Rio de Janeiro state are included. At *municipal level*, all 158 neighborhoods of the Rio de Janeiro city were considered. Finally, at the *local level*, all 652 census sectors, from two RAs of the Rio de Janeiro city (*i.e.*, Jacarepaguá and Cidade de Deus),were used for the analysis. All these areas have in common a high incidence of leptospirosis during the studied period (*i.e.*, from 1996 to 1999). Rio de Janeiro state has one of the highest leptospirosis incidences in Brazil, while the city of Rio de Janeiro has largest number of leptospirosis cases among the municipalities of the state, and Jacarepaguá and Cidade de Deus administrative regions have highest leptospirosis occurrence in Rio de Janeiro city.

### 2.2. Leptospirosis Incidence Data

As leptospirosis is a disease of compulsory notification in the Brazilian Health system (SUS), we obtained the information about its incidence from the National System of Notifiable Diseases [16]. All notified cases, suspected and confirmed, were used to calculate the incidence rates, defined as the ratio between number of cases and population of an area, based on demographic census of 2000. When a patient seeking medical advice, with symptoms like fever, headache, muscle pain, nausea, and reported to be exposed directly or indirectly to water (e.g., flooding, rivers, lakes, *etc.*), medical doctors report it as a suspected case of leptospirosis and recommend further investigation. The suspected cases are confirmed by serological analysis in reference laboratories of SUS [17].

### 2.3. Environmental and Socioeconomic Data

The environmental and socioeconomic information was gathered from several different sources such as: Brazilian Institute of Geography and Statistics [18], Rio de Janeiro Center of Information and Data, CIDE [19] and Pereira Passos Institute-IPP [20]. These data used for this study were obtained from a census survey of 2000 and a satellite image of 2001. It is believed that time difference of these different sources would not bias the analysis because urban studies showed that there is significant lag between urban interventions and changes in the life conditions of a given population, in this case, a change in the leptospirosis incidence. In addition, census occurs every 10 years in Brazil, so the 2000 census is the closest to the study period (1996–1999). Specifically for land use analysis, it was used the municipal quality indexes (IQM) from FCIDE for the Rio de Janeiro state and from IPP for the city of Rio de Janeiro and its RAs of Jacarepaguá and Cidade de Deus. Both CIDE and IPP used Landsat 7 imagery of 2001 to define land use categories. The categories that are deemed relevant concerning leptospirosis occurrence were: urban area, urban area not consolidated, cropland/pasture/grassland and forestland.

We chose to study the data from 1996 until 1999, because we wanted to test if the same environmental and socioeconomic indicators would correlate with leptospirosis incidence during epidemic and endemic periods at the three geographical scales. The epidemic period is represented by the year 1996, because there was a heavy rainy period which resulted in a significant increase in leptospirosis cases (*i.e.*, 2042 cases) in Rio de Janeiro state, compared to the usual average of 500 cases per year registered in the following years (from 1997 until 1999), which represented the endemic period. Also, this choice allowed us to take into account the rainfall data in the study, at least indirectly, because this environmental information was not available for three geographical scales chosen. Besides, another environmental indicator commonly associated with leptospirosis cases, which was not available for state, municipal and local levels, is the presence of rats. Thus, we also assessed it indirectly by garbage collection data.

After obtaining the environmental, demographic and health raw data, databases were generated with specific socioeconomic, environmental, epidemiological indicators at each geographical scale for the study period. The calculation of each indicator is given in Table 1.

### 2.4. Geoprocessing Techniques

Remote sensing techniques and geographic information systems (GIS) were employed in this study. The ArcGis 8.3 software was used at the Georeferencing Laboratory of the Oswaldo Cruz Foundation (FIOCRUZ). To analyze the data, digital maps were used at different units of aggregation associated with their respective geographical scales. For example, all municipalities are shown at Rio de Janeiro state level, each and every neighborhood is depicted at Rio de Janeiro city level and every census sectors are displayed at local level. It was necessary the use of a georeferencing program developed by [21] for the adequate use of all notified cases of leptospirosis at Rio de Janeiro city level, as well as, at the RA level. At the Rio de Janeiro state level, the notified leptospirosis cases were located using municipalities standards codes defined by the Computer Department of the Brazilian Health System (DATASUS). The cases were located at the patients’ municipality of residence.

The GIS was used to associate environmental data maps with the chosen aggregation unit maps at the three geographical scales (state, municipal, and local levels). Unfortunately, these maps limits do not match any Brazilian political boundaries. Therefore, to be able to calculate socioeconomic, environmental and health indicators, such as rates, proportions, and incidences, we have to assume these variables at specific political boundaries, defined as the data aggregation units, which have population information necessary for indicator estimates. These geographical scales and units of aggregation are described in Table 1.

In this context, where the environmental variables (e.g., proportion of areas prone to flooding, altitudes and land use categories) do not overlay with the aggregation units or political boundaries, GIS data manipulation was needed. Only after these operations, it was possible to calculate the indicators used in the statistical tests (*i.e.*, correlations and regressions).

### 2.5. Statistical Analysis

Moran’s index was calculated, using the ArcInfo 8.3 software, to test if there were spatial autocorrelation in the data. Since it showed low autocorrelation both in the epidemic and endemic periods at the three geographical scales, non-parametric Spearman’srank correlation tests were performed between leptospirosis incidence rates and each socioeconomic and environmental indicators. A significance level of 0.02 was chosen for this study because it was considered the best fitted for our study objectives and the nature of data used in the analyses, as described elsewhere [22]. All analyses were carried out with the Statistical Package for the Social Sciences (SPSS) version 10 (SPSS, Inc., Chicago, IL, USA). This test was used to analyze the particular influences of each risk factor studies socioeconomic and environmental variable in leptospirosis incidence.

**Table 1:** Socioeconomic and environmental indicators at three geographical scales and their respective units of aggregation.

Indicators	Geographical Scale (Unit of Aggregation)			
	a. State (municipalities within the Rio de Janeiro state; n = 92)	b.b. Municipal (Neighborhoods in the Rio de Janeiro city; n = 158)	c. Local (Census Sectors of Jacarepaguá and Cidade de Deus; n = 652)
1	Incidence rate of leptospirosis (SINAN and Census IBGE)	Incidence rate of leptospirosis (SINAN and Census IBGE)	Incidence rate of leptospirosis (SINAN and Census IBGE)
2	Altitude classification (FCIDE)	Proportion of areas prone to flooding (IPP)	Proportion of areas prone to flooding (IPP)
3	Proportion of households connected to water systems (Census IBGE)	Proportion of households connected to water systems (Census IBGE)	Proportion of households connected to water systems (Census IBGE)
4	Proportion of households connected to sewage systems (Census IBGE)	Proportion of households connected to sewage systems (Census IBGE)	Proportion of households connected to sewage systems (Census IBGE)
5	Proportion of households with at least one bathroom (Census IBGE)	Proportion of households with at least one bathroom (Census IBGE)	Proportion of households with at least one bathroom (Census IBGE)
6	Proportion of households with systematic garbage collection (Census IBGE)	Proportion of households with systematic garbage collection (Census IBGE)	Proportion of households with systematic garbage collection (Census IBGE)
7	Proportion of population living in slum areas (Census IBGE)	Proportion of population living in slum areas (Census IBGE)	Proportion of population living in slum areas (Census IBGE)
8	Proportion of residents with at least a high school degree (Census IBGE)	Proportion of residents with at least a high school degree (Census IBGE)	Proportion of residents with at least a high school degree (Census IBGE)
9	Population density (Census IBGE and basemap)	Population density (Census IBGE and basemap)	Population density (Census IBGE and basemap)
10	Proportion of land use (Iqm/CIDE): urban area, urban area not consolidated, cropland/pasture/grassland and forestland	Proportion of land use (IPP): urban area, urban area not consolidated, cropland/pasture/grassland and forestland	Proportion of land use (IPP): urban area, urban area not consolidated, cropland/pasture/grassland and forestland
11	Interaction between indicators	Interaction between indicators	Interaction between indicators
Indicative map	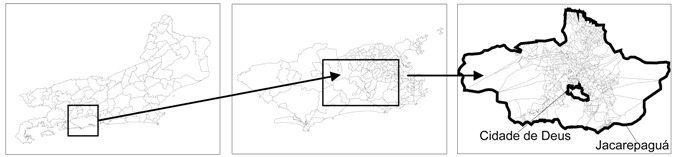			

### 2.6. Indicator Definitions:

(1-a, b, c) (Number of Leptospirosis Cases (SINAN)/population (IBGE)) ×100,000 state level-unit of aggregation municipality; ×10,000 municipal level-unit of aggregation neighborhoods and ×1000 local level-unit of aggregation census sectors. Incidence rate calculations were different among three geographical scales due to large variation in the population at different units of aggregation.
(2-a) Iqm Table with the altitude (m) of each municipality—2003 (FCIDE)
(2-b) Intersection between neighborhood map and map of areas prone to flooding using SIG software ×100
(2-c) Intersection between maps of census sectors of Jacarepaguá and Cidade de Deus RAs and map of areas prone to flooding using SIG software × 100
(3-a, b, c) (Number of households connected to water systems/Total number of households) × 100
(4-a, b, c) (Number of households connected to sewage systems/Total number of households) × 100
(5-a, b, c) (Number of households with at least one bathroom/Total number of households) × 100
(6-a, b, c) (Number of households with systematic garbage collection/Total number of households) × 100
(7-a, b, c) (Total number of people living in slums/Total Population) × 100
(8-a, b, c) (Number of residents with at least a high school degree/Total number of residents) × 100
(9-) Population (IBGE Census of 2000)/Area (Km^2^) for municipalities, neighborhoods and census sectors.
(10-a, b, c) From Landsat images of 2001, CIDE and IPP calculate Iqm for Rio de Janeiro state and Rio de Janeiro city respectively. An operation that intersects between aggregation units used in this work and the land use classifications was performed. Thus polygons with the classification of different land uses within each unit of aggregation was created. Subsequently the ratio of the area of different land uses was calculated: municipalities of the state of Rio de Janeiro; neighborhoods in the city of Rio de Janeiro and the census tracts of the administrative region of Jacarepaguá.

## 3. Results and Discussion

### 3.1. Leptospirosis Incidence Results

**Figure 1:**
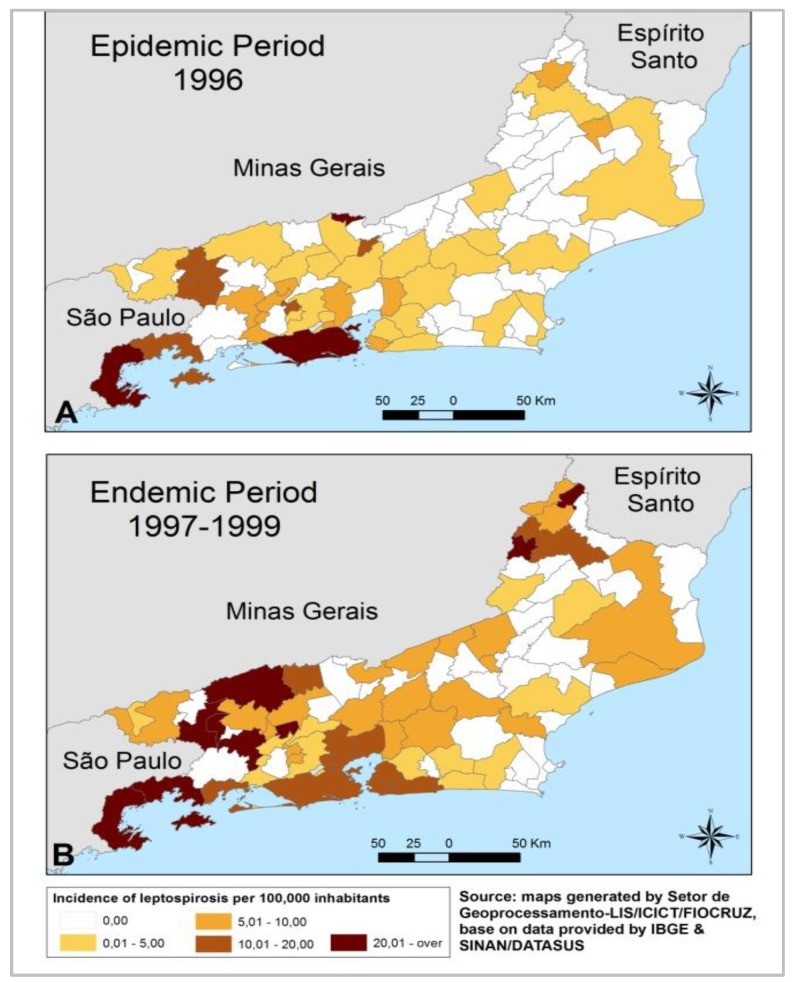
Leptospirosis incidence rates in the municipalities of Rio de Janeiro state during epidemic (**A**) and (**B**) endemic periods, respectively.

Leptospirosis incidence rates in the Rio de Janeiro state during epidemic and endemic periods are shown in Figure 1A,B respectively. We can observe the highest rates in the Rio de Janeiro and Parati municipalities during the epidemic period. While in the endemic period, the Parati rate remains high together with other municipalities of the northwestern region of the state in the Paraíba river valley (close to Minas Gerais state border). It should be noticed that total number of leptospirosis cases in Rio de Janeiro state in 1996 (epidemic period) was the highest in the last 20 years, even though the incidence rates were lower for most of the municipalities in the state. This paradox is explained by the extremely high number of cases in the municipality of Rio de Janeiro which concentrates a large portion of the state population.

**Figure 2:**
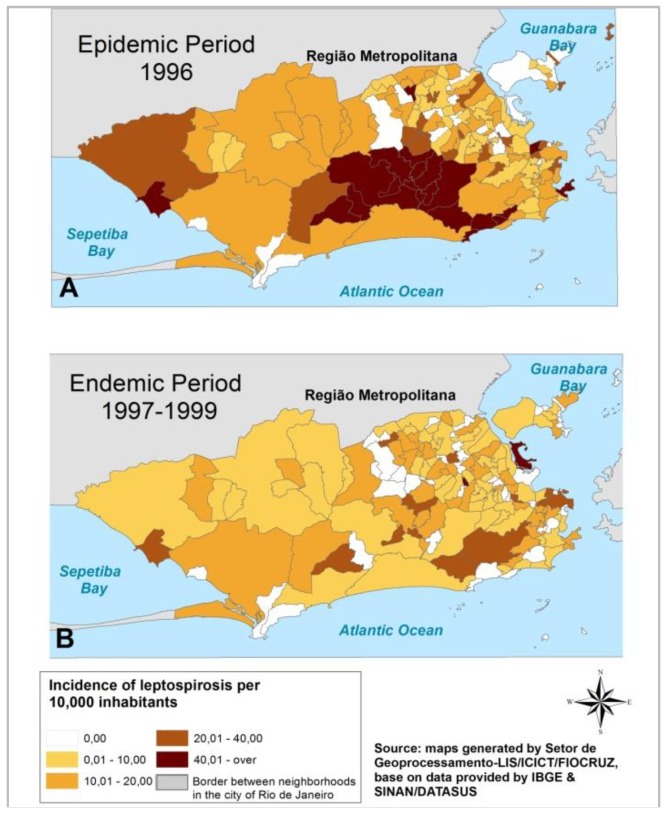
Leptospirosis incidence rates in the neighborhoods of Rio de Janeiro city during epidemic (**A**) and (**B**) endemic periods, respectively.

In the Rio de Janeiro municipality, during the epidemic period (Figure 2A), the neighborhoods with the higher incidence rates were in Jacarepaguá along with some other neighborhoods close to it in the west part of the city (e.g., Camorim, Vargem Pequena, Itanhangá, São Conrado, Joá, Rocinha, and Gávea) and a few other neighborhoods scattered throughout the city (e.g., Urca, Santo Cristo, Barros Filho, and Sepetiba). Whereas, during the endemic period, Figure 2B showes lower incidence rates, with only two neighborhoods in the north part of the city having a high incidence (e.g., Cidade Universitária and Abolição). It should be pointed out that the fast growing axis of Rio de Janeiro city is towards the west and that the northern part of the city is the poorest in the metropolitan area and densely populated.

**Figure 3:**
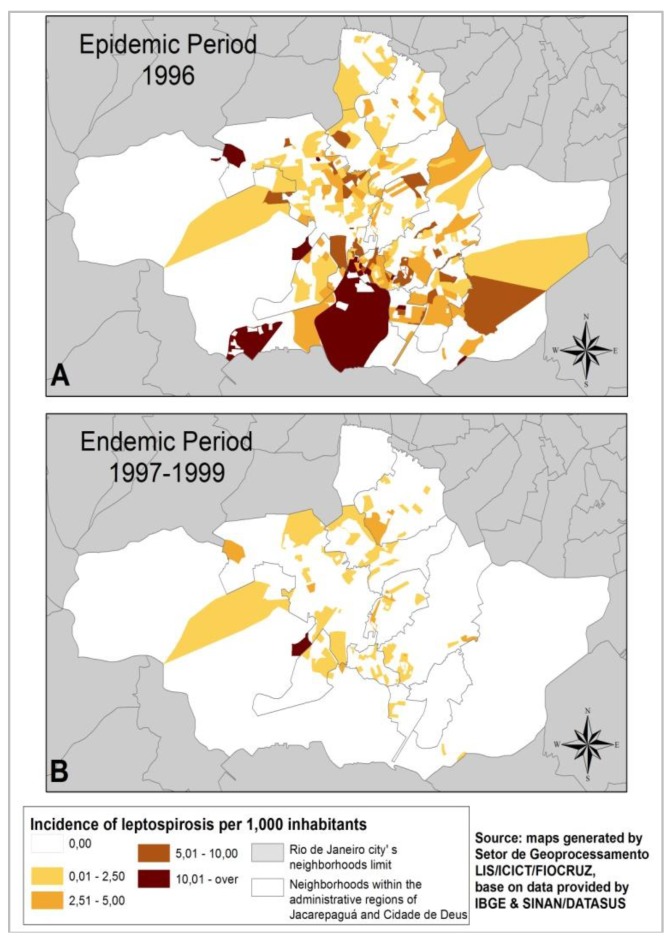
Leptospirosis incidence rates in the census sector of the Jacerapaguá and Cidade de Deus RAs during (**A**) epidemic and (**B**) endemic periods, respectively.

During the epidemic period (Figure 3A), leptospirosis incidence rates in the census sectors of Jacarepaguá and Cidade de Deus neighborhoods did not show any clear spatial trend, but rather a diffuse distribution pattern, although, it was possible to identify a high incidence rate at the Cidade de Deus neighborhood and other few scattered areas. During the endemic period, Figure 3B shows only one hot spot.

Next, the correlation results between incidence rate of leptospirosis and socioeconomic and environmental indicators during the epidemic and endemic periods, for each geographical scale, are presented (Table 2 and Table 3).

**Table 2:** Results of non-parametric Spearman’s rank correlation tests within the epidemic period (1996) at the three geographical scales.

	State Level		Municipal Level		Local Level	
Indicators	Correlation Coefficient	*p*-value	Correlation Coefficient	*p*-value	Correlation Coefficient	*p*-value
Sanitation						
Proportion of households supplied with water	0.241	0.022	−0.204 ^*^	0.010	−0.001	0.975
Proportion of households connected to sewage	0.218	0.038	−0.114	0.153	−0.067	0.085
Proportion of households with garbage collection	0.287 ^*^	0.006	−0.071	0.373	−0.046	0.239
Poverty						
Proportion of population living in slums	0.429 ^*^	0.000	0.082	0.303	−0.180 ^*^	0.000
Proportion of households with at least one bathroom	0.146	0.169	0.017	0.833	−0.029	0.467
Proportion of householders with complete High School	0.211	0.044	−0.083	0.299	−0.010	0.791
Environment						
Altitude/flooding area	−0.04	0.700	0.046	0.566	0.142 ^*^	0.000
Population density	0.350 ^*^	0.001	−0.045	0.577	−0.152 ^*^	0.000
Proportion of urban use	0.387 ^*^	0.000	−0.254 ^*^	0.001	0.033	0.405
Proportion of non-consolidated urban use	-----------	---------	0.323 ^*^	0.000	0.002	0.966
Proportion of field/pasture/anthropogenic use	−0.09	0.393	0.162	0.042	---------	--------
Proportion of rural use	−0.16	0.133	−0.002	0.978	−0.044	0.264

^*^ Correlation is significant at the 0.02 level (2-tailed); Only the incidence rates refer to 1996, the other variables refer to 2000.

**Table 3:** Results of non-parametric Spearman’s rank correlation tests within the endemic period (1997–1998) at the three geographical scales.

	State level		Municipal level		Local level	
Indicators	Correlation Coefficient	*p*-value	Correlation Coefficient	*p*-value	Correlation Coefficient	*p*-value
Sanitation						
Proportion of households supplied with water	0.268 ^*^	0.01	−0.156	0.050	−0.017	0.671
Proportion of households connected to sewage	0.141	0.183	−0.182 ^*^	0.022	0.008	0.832
Proportion of households with garbage collection	0.162	0.126	−0.222 ^*^	0.005	0.003	0.932
Proportion of population living in slums	0.484 ^*^	0.000	0.234 ^*^	0.003	−0.067	0.086
Proportion of households with at least one bathroom	0.256 ^*^	0.014	0.244 ^*^	0.002	−0.028	0.476
Proportion of householders with complete High School	0.346 ^*^	0.001	−0.278 ^*^	0.000	−0.026	0.505
Poverty						
Environmental						
Altitude/flooding area	−0.005	0.959	0.130	0.104	0.014	0.719
Population density	0.253 ^*^	0.015	0.111	0.164	−0.095 ^*^	0.015
Proportion of urban use	0.309 ^*^	0.003	−0.065	0.416	0.013	0.733
Proportion of non-consolidated urban use	-----------	---------	0.120	0.133	0.011	0.775
Proportion of field/pasture/anthropogenic	−0.099	0.349	0.169	0.034	-----------	---------
Proportion of rural use	−0.257 ^*^	0.014	0.115	0.150	0.01	0.802

^*^ Correlation is significant at the 0.02 level (2-tailed); Only the incidence rates refer to 1996, the other variables refer to 2000.

#### 3.1.1. State Level

At this geographical scale, the aggregation unit used was Rio de Janeiro state municipalities. Statistical tests showed that the indicator with the highest correlation with leptospirosis incidence rate, during both endemic and epidemic periods, was the proportion of population living in slums. During the epidemic period, urban land use, population density, and the proportion of households with garbage collection were also positively correlated with leptospirosis incidence rate.

During the endemic period, the indicators: proportion of residents with at least a high school degree and urban land use were positively correlated with leptospirosis incidence and finally, an inverse association was observed between incidence and the proportion of rural use.

#### 3.1.2. Municipal Level

At this geographical scale, the aggregation units are the neighborhoods of the city of Rio de Janeiro. In general, the indicators showed different correlations in the epidemic and endemic periods. During the epidemic period, the indicators that showed the highest correlations with leptospirosis incidence were urban land use (negative) and non-consolidated urban land use (positive).

During the endemic period, the indicators showing the highest correlations with leptospirosis incidence were those associated to poverty, *i.e.*, negatively correlated education and sanitation conditions: the proportion of residents with at least a high school degree, proportion of households connected to a sewage system, the proportion of households with systematic garbage collection and proportion of households supplied with water. 

#### 3.1.3. Local Level

At this scale, the aggregation unit used was the census sector, which is the basic territorial unit used for data collection in Brazilian census surveys. As this is a relatively small geographical area, with a smaller population compared to other aggregation units used in this study, it allows us to analyze the socioeconomic indicators in more detail. However, it also impacts the leptospirosis incidence rates, because small populations generate instabilities in the calculation of incidence rates [23].

The indicators that showed higher negative correlations within the epidemic period were the proportion of population living in slum areas and population density; while altitude and the proportion of area prone to flooding were positively correlated with leptospirosis incidence. 

During the endemic period, the only indicator having a negative significant correlation (*p* < 0.02) was population density. These weak correlation values may be explained by the small number of cases and the statistical instability generated by the small population living in the census sectors. Even if we put together the cases from three endemic years, there were only 73 cases for the whole area at the local scale. Therefore, correlations are more scattered and the indicators differ according to the geographical scale used (Figure 3B).

### 3.2. Discussion

Correlation analyses, for epidemic and endemic periods at the three studied scales (*i.e.*, state, municipal and local levels), showed different results. The diverse outcomes seemed to be influenced by the geographical scale considered. The associations observed at one scale were not present at another one. Although, under some conditions, leptospirosis incidence displayed significant correlation with well-known variables (*i.e.*, poverty indicators), corroborating results from other studies on the occurrence of leptospirosis in urban poor areas [1,2,3,4,5,24], that were not always the case. The geographical scale, at which the correlations showed results closer to what was expected according to the literature, was the municipal level, where neighborhoods was the aggregation unit used. The utilization of census sectors as the unit of aggregation at local level scale resulted in smaller number of cases, which might be insufficient to guarantee the needed stability to calculate incidence rates adequately.

The observed correlations between higher coverage of sanitation services and the incidence of leptospirosis at the state scale, differently from what have been reported in the literature [2,3,8,9,10,13], may be related with a higher incidence of leptospirosis in large urbanized cities with bigger population density. They would have a higher proportion of the population with water supply, sewage system, garbage collection, households with at least one bathroom, and residents with at least a high school degree. At this scale, urbanization indicators may be positively associated with leptospirosis incidence. This was more evident during the epidemic period, when large cities, such as Rio de Janeiro, have high incidence rates, that cause misleading positive correlations with sanitation service coverage. However, it is important to note that the proportion of population living in slum areas was always positively correlated with leptospirosis incidence at this geographical scale, indicating the vulnerability of this portion of the population in large cities.

Other data would be necessary to analyze local risks properly, such as the maps of slums, of the topography, of rivers and lakes, to be able of performing spatial queries in a GIS environment and to classify slum areas into the specific categories (e.g., raised, plains, and riverbanks); because the geographic location and spatial configuration of the slums can facilitate or hinder leptospirosis transmission. We chose not to use these data because they were not available for all slums in the studied area.

Regarding land use, we noted out a positive correlation with urban areas, a result that corroborates the reports by other authors in the literature [1,2,25]. In addition, it is known that leptospirosis transmission in the state of Rio de Janeiro occurs mainly in urban areas, or that the notification and investigation of cases in rural areas are not as effective.

Although the correlation coefficients obtained in this study were not generally elevated, they were significant at the 0.02 level. The small observed coefficients might be explained, at least partially, by the fact that the data sources used in this study have different time frames. For example, the leptospirosis data are from 1996 to 1999, while the socioeconomic and environmental data are from 2000 to 2001.

The indicator regarding the proportion of population living in slums may cause some inconsistencies in the results. Although, it was positively correlated with leptospirosis incidence at state level in both the epidemic and endemic periods, it was significant at the municipal level only in the endemic period, and it was negatively correlated at the local level in the epidemic period. This might have happened because of some bias in the data. For example, at the local level, some areas, such as Cidade de Deus, which has an urban structure and a landscape that is typical of a slum area, are not classified as slums by IBGE, because most people have a house ownership document and there are some established sanitation services. Thus, an important part of the population living in slums at the local level was underestimated. At the municipal scale, there were no leptospirosis notifications in the non-consolidated urban use areas, which is surprising since there were notifications in those areas when other geographical scales were used. The probable reason is the different resolutions of the satellite images of the Rio de Janeiro state and Rio de Janeiro city, which may have discarded this land use category at municipal level.

It can be noticed that the central area of Rio de Janeiro city, known as Baixada de Jacarepaguá, located between two mountain ridges, concentrates the highest incidences of leptospirosis. It has environmental features that increase its vulnerability when compared to the rest of the city. Since it is located in a valley surrounded by high forested areas (*i.e.*, Pedra Branca and Tijuca), it is a region prone to flooding due to tropical rainfall [26]. Indeed, the indicator regarding altitude at the state level had a negative correlation, although not statistically significant, in both periods. This result followed the rationale that the higher a city’s altitude is, the lower is the possibility of flooding when there is heavy rainfall, and consequently, the lower is the risk of leptospirosis.

Our study aimed to identify what indicators are more important to explain the leptospirosis incidence at different geographic scales. The literature review conducted in this study showed that, at the global scale, the development level of countries determines the distribution of leptospirosis [2]. In turn, at the regional scale, weather, types of agriculture production and land use in rural and urban areas, respectively, are the main determinants [27,28]. At the local scale, sanitation conditions are the most important determinants. The absence of adequate sanitation services, such as lack of sewage systems or suitable trash disposal, favor a local increase in the number of rodents, which in turn can cause the spread of *Leptospira* and the consequent high leptospirosis incidence [4,5]. 

It should be emphasized, however, that one isolated indicator cannot explain the incidence of a complex infectious disease, such as leptospirosis, fully. Confounding factors should also always be considered. For instance, we observed some unexpected positive and significant correlations with some indicators in this study, when comparing the epidemic and endemic periods. During the epidemic period, water supply, as well as, sewage and garbage collection, showed positive correlations when negative correlations were expected (*i.e.*, a better sanitation infrastructure should reduce the incidence of leptospirosis, especially with regard to waste collection, since areas with accumulation of garbage are prone to be infested with rats, which are usual leptospirosis vectors). 

**Figure 4:**
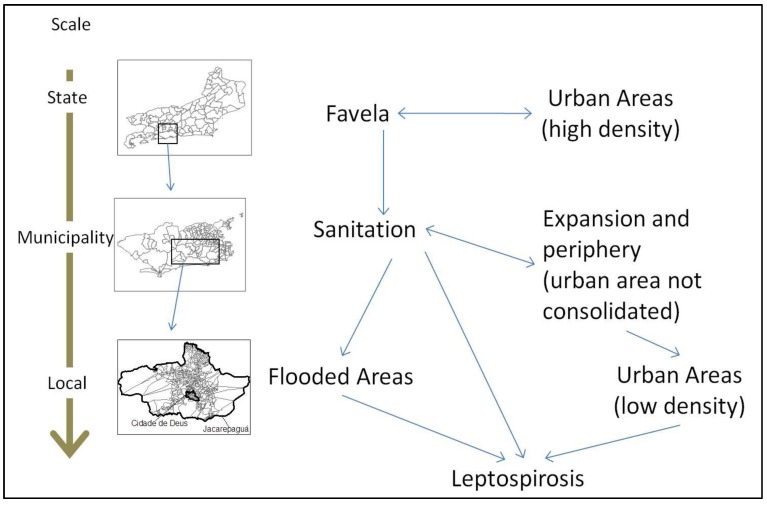
Multiscale model for leptospirosis determinants analysis.

Caution should be used when studying associations between leptospirosis risk and isolated variables. A combination of factors is needed to increase its risk. For instance, the regions with higher rainfall, which would be at a higher leptospirosis risk, are not necessarily the regions where there is a higher incidence of this disease [24]. In a similar way, poverty can be a proxy for leptospirosis incidence. However, it needs to be specific poor conditions characterized by widespread sanitation problems, agglomeration of people, and susceptibility to flooding. Therefore, an interdisciplinary approach is necessary to address a multifactorial disease, like leptospirosis, as shown in Figure 4.

## 4. Conclusions

Although, there are few empirical studies about this concept, the choice of the geographical scale of a study is among the first methodological issues that must be addressed in a research. Different data (e.g., environmental, socioeconomic, epidemiological, *etc.*) are produced and aggregated using different scales, usually associated with political or administrative divisions. However, the studied phenomena, in general, are not restricted to any of these boundaries. Therefore, it is essential to be able to combine, visualize and analyze the data at different scales, and the decision of which geographical scale should be used is not a trivial task.

Indeed, the results of this study suggested that outcomes from ecological studies, looking for an association between environmental or socioeconomical factors and disease determinants of a multifactorial disease, such as leptospirosis, might be influenced by the geographical scale, unit of data aggregation and period chosen for the analysis. One geographical scale, using a certain data aggregation unit, may show some clear associations that can be hidden in another geographical scale. No geographical scale should be regarded as definitive, it must be compatible with the studied phenomenon in order to highlight its functioning, causes and consequences.

The well-known association between leptospirosis incidence and lack of sanitation was evident at the municipal level of this study, but it was absent at the other two scales used (*i.e.*, state and local levels). Actually, at the state level, the data seems to indicate that better sanitation services (*i.e.*, Proportion of households supplied with water, connected to sewage and with garbage collection) are associated with higher incidence of leptospirosis. In fact, these significant positive correlations are misleading because, at this geographical scale, where different municipalities are compared, they reflected mainly their different degrees of urbanization. Large urbanized municipalities, which have generally better sanitation services, have more leptospirosis cases, as positive associations between leptospirosis incidence and population density or proportion of urban use indicated. At the municipal level, where different neighborhoods of a city are compared, the worst sanitation services of poor, less urbanized, areas are associated with leptospirosis cases as indicated by their negative significant correlations. Thus, the same indicator (e.g., proportion of urban use) can be either positively or negatively correlated with leptospirosis incidence depending on the geographical scale used (e.g., state or municipal levels).

On the other hand, when looking at the local level, where the census sectors of one neighborhood are compared, it was not possible to see any association with sanitation or most of the other studied factors. A possible explanation is the reduced number of leptospirosis cases in each census sector, which cause instabilities in incidence rates calculations. In addition, sanitation services may not vary significantly among the studied sectors, which represent a relatively small geographical area. Probably, some other detailed data would be necessary (e.g., topographical maps of slums) to evaluate associations between leptospirosis incidence and socioeconomical or environmental factors, at this geographical scale, properly. Besides some calculated indicators might be underestimated (e.g., portion of the population living in slums) at this reduced scale.

Hence, one geographical scale was more adequate to show associations between leptospirosis incidence and a group of factors. In this study, we used three main groups: sanitation, poverty, and environment. While sanitation factors’ associations, as well as poverty ones, were more evident at the municipal level, environmental factors significant correlations were more common at the state level. A remarkable exception among environmental factors studied, was the association of leptospirosis and flooding area, which was significant at the local level only. This might be related with availability, or even accuracy, of specific variable. For instance, flooding areas are generally used in intra-urban local analyses, where it can be defined easily. Yet, at regional analysis, it is difficult to delimit these areas, and proxies, such as altitude information, must be adopted. This means that differences in surface size imply quantitative and qualitative differences concerning the phenomena. Thus, we recommended that studies shall gather information of different variables at appropriated scales, due to inability to visualize some geographic features at some scales.

To conclude to assess leptospirosis risk for a specific population properly, health surveillance should take into account not only typical socioeconomical and environmental factors, but also the context in which they are analyzed, which included the geographical scale, aggregation unit and period under analysis. The risks associated with each factor may vary in magnitude and direction depending on the combination of these issues. For instance, poor populations, living in peripheral areas, with precarious sanitation services, of large, densely populated cities, could be at greater risk for leptospirosis transmission. Another combination of factors that could produce an increase in leptospirosis transmission risk is extensive flooding areas near the slums of big cities. Thus, risks have a multifactorial and multilevel nature. One factor alone is not sufficient to increase risk, and a combination of factors at a specific geographical scale are necessary. For example, at the geographical scale of Rio de Janeiro state, we may claim that the socioeconomic and environmental indicators that must be used to indicate the vulnerability of a municipality to the occurrence of epidemics of leptospirosis are the proportion of population living in slums, population density, and urban use.
